# Nurse leaders’ perceptions of future leadership in hospital settings in the post-pandemic era: a qualitative descriptive study

**DOI:** 10.1108/LHS-05-2023-0032

**Published:** 2023-09-28

**Authors:** Eeva Vuorivirta-Vuoti, Suvi Kuha, Outi Kanste

**Affiliations:** Research Unit of Health Sciences and Technology, Faculty of Medicine, University of Oulu, Oulu, Finland

**Keywords:** COVID-19 pandemic, Leadership, Nursing, Future, Post-pandemic era, Hospital, Content analysis, Qualitative study

## Abstract

**Purpose:**

Coronavirus disease (COVID-19) has challenged leadership in hospitals worldwide. The experiences of leadership during the pandemic changed leadership significantly. This study aims to describe nurse leaders’ perceptions of what future leadership in hospital settings in the post-pandemic era needs to be like.

**Design/methodology/approach:**

A qualitative descriptive study was used. A total of 20 nurse leaders from the Finnish central hospital were interviewed from June to October 2021. The data were analysed using inductive content analysis.

**Findings:**

The analysis revealed five main categories describing nurse leaders’ perceptions of future leadership in hospital settings in the post-pandemic era: digitalisation and hybrid working culture, development of sustainable working conditions, moving smoothly to the post-pandemic era, dissolution of traditional regimes of organisation and flexibility in leadership.

**Practical implications:**

In the post-pandemic era, the constantly changing demands and challenges currently facing healthcare systems have significantly increased the complexity of hospital organisations. This requires critical evaluation and change to traditional leadership. Enhancing flexibility and authenticity in leadership, strengthening competencies, implementing a wide range of digital resources and increasing the appeal of the nursing profession to build the next generation of nurses – all of these are needed to provide sustainability in future healthcare.

**Originality/value:**

The results identify the critical points of leadership that need to be developed for future challenges and for maintaining a sufficient supply of qualified professionals. Acting on this information will enhance flexibility in organisations and lead to acceleration of changes and the development of new kinds of leadership in the future

## Introduction

Coronavirus disease (COVID-19) is an infectious disease caused by the SARS-CoV-2 virus. The World Health Organization (WHO) declared COVID-19 to be a global pandemic on 11 March 2020 (WHO, 2020a). This global pandemic has extensively challenged healthcare services and leadership; however, the impact of the pandemic on nurse leaders’ work has been especially significant (Raso *et al.*, 2021).

The COVID-19 pandemic is still, three years after its outbreak, influencing the operation of healthcare services worldwide. Distress among healthcare professionals increased during the pandemic (Hossny *et al.*, 2022) and intensified job turnover intentions (Haq *et al.*, 2022). Simultaneously, the global shortage of nursing professionals created an enormous challenge for healthcare, and efforts are required to develop leadership that will work effectively to build a more sustainable workforce (WHO, 2020b).

Nurse leaders’ work involves several and often simultaneous activities related to the organisation of each unit’s daily work. Managing human resources, supporting professionals, developing, planning (Nurmeksela *et al.*, 2020; Sveinsdóttir *et al.*, 2018) and evaluating operations (Nurmeksela *et al.*, 2020) are important parts of nurse leaders’ work. In addition, they should ensure all professionals’ well-being and a high-quality working environment (Nurmeksela *et al.*, 2020) as well as oversee communication and collaboration within the unit and organisation (Nurmeksela *et al.*, 2020; Sveinsdóttir *et al.*, 2018). Nurse leaders’ actual involvement in clinical nursing varies depending on the work unit (Sveinsdóttir *et al.*, 2018), but generally, nurse leaders rarely participate in clinical work (Nurmeksela *et al.*, 2020).

Nurse leaders’ competencies include the skills, knowledge and abilities that are needed to organise safe, efficient, equitable and patient-centred care [American Organization of Nurse Executives (AONE), 2015]. AONE divides the competencies of nurse leaders into three domains: the science, the leader within and the art. The first domain includes competencies that are needed in managing the business, such as financial skills, human resource management, technology and strategy. The second domain includes aspects related to self-leadership and growth. The third domain focuses on leading people, for example, to build relationships and encourage diversity. González‐García *et al.* (2021) have identified 53 different leadership competencies, which are further divided into six domains: management, communication and technology, leadership and teamwork, knowledge of the healthcare system and nursing knowledge and personality.

During a crisis such as a pandemic, the demands involved in nurse leaders’ work differ from those of a normal situation. There will be an increased emphasis on leadership exhibiting strong self-confidence, responsibility and resilience when facing the relentless challenges posed by such a crisis. Motivation, communication, decision-making, coordination and reorganisation skills are highlighted as necessary (Van Wart and Kapucu, 2011).

During the COVID-19 pandemic, the leaders of healthcare professionals were forced to quickly change their approach to how care was provided as they adapted to a situation that was radically different from what they had experienced prior to the pandemic. One of the most significant changes was the implementation and expansion of remote leadership (Ameel *et al.*, 2022). Simultaneously, the allocation of human resources was reconsidered and organised in a different way (Hossny *et al.*, 2022), and novel communication and networking (Freysteinson *et al.*, 2021; Morse and Warshawsky, 2021) were emphasised. The leaders’ onsite presence, motivational and psychological support skills (Freysteinson *et al.*, 2021; Hossny *et al.*, 2022), effective communication (Hossny *et al.*, 2022) and trustworthiness (Freysteinson *et al.*, 2021) became more important than ever before, although remote leadership expanded. Authentic leadership proved indispensable during the pandemic (Raso *et al.*, 2021).

Nursing leadership and competencies develop constantly over time as organisations change (Vasset *et al.*, 2023). Studies have envisioned what future leadership could be foreshadowing the demands likely to be faced by nursing leadership. For example, Sanford and Janney (2019) recommended updates to AONE’s leadership competencies based on future healthcare scenarios. In addition, studies have focused on examining leaders’ perceptions of future leadership (Gjellebæk *et al.*, 2020; Nurmeksela *et al.*, 2021; Pihlainen *et al.*, 2019) and describing the leadership competencies needed during and after the pandemic (Dirani *et al.*, 2020; Morse and Warshawsky, 2021; Okoli *et al.*, 2022). These studies confirm the long-lasting influence that the pandemic has had on leadership.

In the future, leadership in hospitals will probably be less hierarchical, and the focus of development will be on improving the attractiveness and effectiveness of nurse leaders’ work and organisations (Nurmeksela *et al.*, 2021). Leadership in the hospital setting will be more strategic and proactive (Nurmeksela *et al.*, 2021; Sanford and Janney, 2019), emphasising changes in leadership and financial competencies (Pihlainen *et al.*, 2019; Sanford and Janney, 2019). Moreover, crisis and ethical leadership will become of greater importance in the future (Morse and Warshawsky, 2021). The use of technological solutions (Dirani *et al.*, 2020; Gjellebæk *et al.*, 2020; Morse and Warshawsky, 2021), supporting the well-being of professionals (Dirani *et al.*, 2020; Morse and Warshawsky, 2021; Nurmeksela *et al.*, 2021; Okoli *et al.*, 2022), and communication interaction along with effective networking skills (Pihlainen *et al.*, 2019; Sanford and Janney, 2019) will also be pivotal in future leadership. In addition, the ability to lead people, allocate human resources (Morse and Warshawsky, 2021; Pihlainen *et al.*, 2019), employ current knowledge (Sanford and Janney, 2019) and develop evidence-based practices will be essential (Morse and Warshawsky, 2021; Nurmeksela *et al.*, 2021). Future leaders should be decisive (Okoli *et al.*, 2022), self-confident and innovative and have good decision-making (Sanford and Janney, 2019), time management (Pihlainen *et al.*, 2019) and digital skills (Gjellebæk *et al.*, 2020). Continuous change in healthcare thus requires leaders with learning abilities (Vasset *et al.*, 2023) and more comprehensive educations (Morse and Warshawsky, 2021).

Leadership in hospital settings has been studied in terms of nurse leaders’ perceptions of the components of their work as leaders *per se*, and experts have provided views on the competencies that are needed. However, limited studies are available on nurse leaders’ perceptions of future leadership, especially in the post-pandemic era. The pandemic changed leadership, particularly in hospital settings, and that change is likely to be permanent. Therefore, there is a great need to explore the lessons that can be learned from the COVID-19 pandemic. Thus, this study describes nurse leaders’ perceptions of what future leadership in hospital settings in the post-pandemic era needs to be like. The research question was: What kinds of perceptions do nurse leaders have of future leadership in hospital settings in the post-pandemic era?

## Methods

### Design and study settings

This study used a descriptive qualitative design that explored and described nurse leaders’ perspectives based on their own lived experiences (Kyngäs *et al.*, 2019). The empirical data were collected through semi-structured interviews (*N* = 20) with frontline nurse leaders and middle managers of one Finnish central hospital with approximately 2,000 healthcare professionals, 389 hospital beds and 15 medical specialties. The study’s target hospital had to deal with a massive influx of COVID-19 patients in the early phase of the pandemic.

### Participants

The participants were selected through purposive sampling and recruited from the hospital via email invitation. The inclusion criteria for the participants were that they had been working in a management position during the COVID-19 pandemic and that they had adequate Finnish language skills to participate in an interview. A total of 48 nurse leaders were invited to participate in the study, and 20 agreed to participate. Of the 48 participants, three did not meet the inclusion criteria, two refused due to busy work situations, and 23 were unreachable despite repeated attempts.

Sixteen (80%) of the participants were frontline nurse leaders, and four (20%) worked in middle management positions. The mean age was 51 years, and the mean leadership experience was 10 years. Four participants had a college degree, seven had a bachelor’s degree, and nine had at least a university master’s degree. Participants worked in different departments, wards and clinics in the hospital: emergency, intensive care, rehabilitation, psychiatric care, obstetrics and gynaecology and paediatrics as well as care service units. Background information was collected through a Webropol survey (Table 1).

### Data collection

This study was part of a larger research project that examined change and crisis management during the COVID-19 pandemic, future leadership after the pandemic, the effects of the pandemic on leaders’ work well-being and social support for leaders in a hospital setting. The interview guide, covering the common themes of the research project and interview questions of this study, is shown in Interview guide for the research project.

Main themes (1–5) of the research project and semi-structured interview questions of this study (3):

1. Changes in leadership during the COVID-19 pandemic in the hospital;
2. Crisis leadership during the pandemic in the hospital;
3. Future leadership after the pandemic in the hospital
  - What did the pandemic teach you about leadership?
  - What kind of effects has the pandemic had on your leadership? What is positive? What is negative?
  - Thinking of the time after the pandemic, how are professionals prepared for a new normal?
  - How do you see leadership after the pandemic, after the state of emergency and digitalisation?
  - What kind of leadership know-how will be emphasised in the future?
  - What kinds of new competencies are leaders going to need?;
4. Effects of the pandemic on leaders’ work and their well-being; and
5. Social support for leaders in the hospital.

Source: Authors’ own work.

The questions used in this study were related to the theme of future leadership. The interview guide was based on the findings of previous research (Karabacak *et al.*, 2011; Van Wart and Kapucu, 2011) and was further developed by a research team of experienced qualitative researchers and experts in nursing leadership. The interview guide was pre-tested by two leaders possessing relevant experience in leadership during the COVID-19 pandemic. Based on these pilot interviews, the number of questions was reduced, and some were reworded to make them more comprehensible. The pre-testing data did not form part of the final data. Data were collected remotely by a well-acquainted research team (*N* = 5) following uniform guidelines from June to October 2021 using Microsoft Teams. The interviews were recorded with permission from the participants. The interviews lasted 79 min on average (ranging from 54 to 109 min).

### Data analysis

The data were analysed using inductive content analysis (Kyngäs *et al.*, 2019). Interviews were transcribed and amounted to 526 pages (12-point Times New Roman font; 1.5 line spacing). The data analysis process began with the selection of the unit of analysis, which was a theme or sentence describing future leadership. The transcribed data were read carefully multiple times, searching for original expressions related to the research question. The original expressions (*N* = 755) were grouped into subcategories (*n* = 26), categories (*n* = 11) and main categories (*n* = 5). An example of the analysis process is presented in supplementary table 1. The data were analysed using NVivo software (NVivo, 2022).

### Rigour

The trustworthiness of a study is based on its credibility, dependability, confirmability, authenticity and transferability (Kyngäs *et al.*, 2019). In our case, credibility was strengthened by an accurate description of the study process and ensuring that data saturation was reached. Data saturation was reached after 17 interviews. Dependability of the study was strengthened by using tables that describe the analysis process transparently. The open codes, categories and analysis were discussed by the research team. Confirmability was ensured by meticulously retaining the connection between the data and results during the analysis process. Authenticity was confirmed with quotations from the original data in findings. Transferability was enhanced by describing the background information of participants and using consolidated criteria for reporting qualitative research (COREQ) to report the findings (Tong *et al.*, 2007). COREQ checklist is presented in supplementary table 2.

### Ethical considerations

The study was conducted with responsibility, integrity, meticulousness and accuracy, following the guidelines of the Finnish National Board on Research Integrity (TENK) and the ethical principles of the World Medical Associations (WMA) Declaration of Helsinki (TENK, 2019; WMA, 2013). This study did not require the Research Ethics Committee’s approval since it did not in any way threaten or affect their physical integrity, expose them to exceptionally strong stimuli, or cause mental harm or threat to their safety (TENK, 2019). Permission to carry out the research was obtained from the target hospital. To give their written informed consent electronically, the participants were informed of the study process and interview content via email. In addition, verbal consent to participate and be audio-recorded was obtained. Also, the voluntary nature of their participation, the possibility of withdrawing at any time and the confidentiality of the data were explained before the interviews (TENK, 2019). The results of the study have been reported with accuracy, following ethical principles and ensuring that participants are not recognisable (WMA, 2013).

## Findings

The analysis revealed five main categories describing nurse leaders’ perceptions of future leadership in hospital settings in the post-pandemic era: 1) digitalisation and hybrid working culture, 2) development of sustainable working conditions, 3) moving smoothly to the post-pandemic era, 4) dissolution of traditional regimes of organisational structure and 5) flexibility in leadership (Table 2).

### Digitalisation and hybrid working culture

According to the analysis, digitalisation and hybrid working culture included two categories: using digitalisation for leadership and developing a hybrid working culture in leadership.

Using digitalisation for leadership included the possibilities of implementing digitalisation and resources needed for implementing it. Implementing digitalisation can increase the effectiveness of operations in hospital settings, for example, by enabling new kinds of procedures in patient care and by both diversifying and equalising services. In addition, the participants noted that it can enhance financial savings and increase the use of scarce resources and time. In particular, digitalisation was viewed as viable means of expediting meetings and improving educational practices. According to the leaders, both digitalisation and its development were quickly adopted during the pandemic and will continue to be used and expanded in the future. Leaders considered digitalisation to be positive:

It might have been noticed, during the pandemic, that digitalisation needs to be brought forward. Don’t know if we would have gone forward in so many things without this pandemic. So, from that aspect, this has been a good thing. (Interviewee 19)

Our analysis shows resources that are needed for implementing digitalisation will need to increase in the future. Resources such as time and equipment are needed for developing and implementing digitalisation and for training professionals. In addition, the successful deployment of digital solutions requires that leaders be aware of the latest technological resources and involved in planning how to best use them. The most essential requirement, according to participants, was enhancing the knowledge and digital competencies of leaders, who need to have education on information technology and the use of digital solutions, as well as knowledge and skills in digital leadership, including the ability to motivate others to adopt and apply digital solutions.

According to our analysis, the development of a hybrid working culture in leadership requires recognition of the possibilities of adapting remote work to leadership and the challenges to leadership presented by a hybrid work approach. Remote leadership expanded significantly during the pandemic, and this is expected to continue in the future, enabling further and better development of hybrid leadership, facilitating leadership of larger and more divergent departments, rationalising the use of time and contributing to the concentration leadership. Thus, remote leadership offers possibilities for organising leadership in new ways:

If I can attend meetings remotely, it allows me to control my time. (Interviewee 13)

The challenges of hybrid work in leadership are related to interaction and the leaders’ accessibility. Leaders perceived that the human aspect of nursing emphasises interaction and communication between people, which is challenging when communicating remotely using online collaboration applications. In addition, the accessibility of leaders is pivotal when working remotely. Overall, the results showed that the ongoing development of a hybrid work culture is needed in the future.

### Development of sustainable working conditions

The development of sustainable working conditions included two categories: enhancing professionals’ work engagement and well-being at work.

Our analysis revealed that enhancing work engagement requires improving work conditions, competence in leading new generations of professional nurses and increasing appreciation of professionals. Improving work conditions requires maintaining a sufficient supply of professionals and increasing the appeal of the nursing profession. Maintaining sufficiency is challenging, and leaders are highly concerned about nurses’ increasing intentions to leave the profession, such as those who are considering changing careers or pursuing further studies, even if they appreciate nursing as a profession. Leaders noted that competence in recruiting is a pivotal part of maintaining sufficiency. They added that the appeal of the nursing profession should be increased by organising work in a meaningful way and reassessing job-related communities and leadership. Accordingly, human resource leadership competencies and policies scheduling, allocation and rewards are important for increasing meaningful work and enhancing work engagement.

According to leaders’ perceptions, leading new generation by listening to, understanding and learning from young professionals was noted as ways to motivate and support work commitment. Leaders should be open to changes in young professionals’ needs, nursing leadership and themselves. In addition, the reputation of a leader or work community will be even more essential in the future, as it will influence workplace selection for young professionals. Leaders underlined the need for a change in leadership when leading the new generation:

Understanding that we lead a new generation, which have worries of their own. Can’t tell them that this is how we have always done things. Maybe, you as a leader, should have understanding that you should change. (Interviewee 9)

The study findings also indicate that professionals should be more appreciated, which would enhance work engagement. They should be rewarded equitably for showing flexibility during the pandemic. Wage levels should be fixed to be commensurate with the number of duties and responsibilities undertaken. In addition, emphasising the positive aspects of nursing in the media was perceived to be essential in the future.

Increasing well-being at work requires constant support for leaders and professionals. According to the analysis, the well-being of nursing professionals and leaders was challenged during the pandemic, and leaders are highly concerned about coping in the future. Leaders reported that the pandemic caused fatigue, anxiety, uncertainty, stress, fear and uncontrollable emotions among nursing professionals. The negative effects of the above, for example, such as the need for more and longer sick leaves, presented major problems during the pandemic and may continue to present difficulties even as the pandemic subsides; therefore, leaders should constantly pursue and support the well-being of professionals. Recognising the challenges and supporting factors, such as offering the possibility for open dialogue, were perceived as ways to help professionals cope:

All that anxiety they [nurses] have been through during this time will fall on them when things get easier, and leaders should be there monitoring how they are doing. (Interviewee 5)

Leaders stressed the importance of peer support for their own well-being. In addition, mentoring, support from superior leaders and debriefing after the pandemic were noted as factors that will support leaders’ well-being in the future.

### Moving smoothly to the post-pandemic era

Moving smoothly to the post-pandemic era included two categories: recovering from the pandemic and anticipating and preparing for future crises.

Recovering from pandemics requires changing to a new normal, assessing and restructuring operations that were affected, and reflecting on how pandemics can be more effectively responded to within the organisation. According to the interviewees, changing to a new normal is challenging to define, and there are differences in how the change should be done. Leaders perceived that the change would happen imperceptibly as the restrictions were eliminated. They also perceived that moving towards a new normal is a change that needs to be led.

Assessing and restructuring operations caused by pandemics are perceived as part of the recovery process. Assessment should be done on prioritising and compressing operations in cooperation within hospitals by evaluating operations affected by the pandemic related to the present situation of the organisation. The operational changes made during the pandemic, which are not seen as necessary for the future, should be eliminated as soon as the situation allows. Some procedures shown to be beneficial, such as effective hand hygiene and cooperation between units, should be retained. Leaders emphasised that reviewing and reflecting on the pandemic’s effects within the organisation is essential. According to leaders, negative experiences and emotions caused by the pandemic should be reflected on communally to support professionals recovering from and processing the psychological burden of the pandemic. Reflecting will also allow leaders to be heard:

Also, comments from leaders, what was good, what was bad, where did we succeed and what should be done differently. (Interviewee 17)

According to our analysis, anticipating and preparing for future crises requires a combination of strategic directions for future crises and strengthening crisis leadership competence. Procedures for dealing with future crises should be developed by involving all professionals working within the hospital and based on the lessons learned from the pandemic. Strengthening crisis leadership competence by increasing education about leadership in crisis situations and regularly reinforcing this education, as well as reviewing and updating crisis management strategies, are the basics of successful preparation:

I hope that we could have regular crisis education in the future to maintain competence to handle crisis to come, even if they are not as big as this one [pandemic]. (Interviewee 13)

### Dissolution of traditional regimes of organisation

The dissolution of traditional regimes of organisational structure included two categories: using shared leadership and enhancing cooperation in the organisation.

Our analysis shows that using shared leadership requires restructuring the hierarchy in an organisation and sharing the responsibilities of leaders. According to the leaders, rigid hierarchical management systems based on positions and regimes do not function well in contemporary organisations. Leaders prefer more modern leadership styles that emphasise co-work instead of top-down ordering. Sharing the responsibilities of leaders includes the constant involvement of leadership to promote joint responsibility within work communities – that is, delegating tasks and duties to co-leaders and professionals. Strengthening the role of the nurse in charge while leaders are absent will also be essential in the future. Leaders emphasised common responsibilities in the future:

Sharing responsibilities. Only one person can’t be responsible. We have to commonly answer of matters of this unit. (Interviewee 7)

Our analysis indicates that enhancing cooperation within organisations requires improving communality in work communities and strengthening interactions within the organisation and environment. The pandemic had a positive impact on communality in the work environment that should be further improved in the future by developing multi-professionality, improving social well-being, teamwork and workplace atmosphere and enhancing communal development. Analysis revealed that enhancing communality requires truly involving professionals in its development by recognising that they have the most updated knowledge of value, encouraging them to be creative in developing new procedures, sharing their ideas and being unprejudiced towards the ideas.

Our findings show the importance of strengthening interaction within the organisation and organisational environment. Strengthening interaction within an organisation requires leaders’ strong cooperation and networking skills and strengthening trust among professions. Top management should show trust in professionals’ competencies. Showing strong trust in middle- and front-line nurse leaders as decision-makers in their own units is also essential. Furthermore, top management should become more closely involved in nursing practice to gain more updated knowledge of basic operations and working conditions in hospitals.

In addition, strengthening interaction within the organisation and environment requires enhancing cooperation with other in-hospital units and healthcare providers outside the hospital. Enhancing cooperation was perceived as pivotal for ensuring adequate resources in the future. Leaders should be more aware of outside threats to organisations and changes in surrounding societies. Leaders should also be able to rationalise the effects of changes and procedures from outside their working environments. According to leaders, dissolution of traditional regimes through effective cooperation could increase flexibility in the use of scarce resources:

We should find out that can we do some things with more agility in cooperation with the city. Join forces and figure out how to use resources in best way. (Interviewee 17)

### Flexibility in leadership

Our analysis revealed that flexibility in leadership included three categories: increasing authentic encounters in leadership, developing strong self-leadership and enhancing agility in an organisation.

Leaders perceived that the pandemic changed leadership and that it would change even more in the future. The pandemic showed that people need to be led with increasing authentic encounters in leadership by enhancing the meaningful presence of leaders. Transparent communication was emphasised as important for the future. Enhancing the meaningful presence of leaders requires human leadership skills and recognising the significance of leadership presence in work communities. In human leadership, the ability to be open-minded and flexible but also explicit and robust in decision-making and actions was underlined as essential. The leaders we interviewed stressed that more authentic and helpful coaching approaches where leaders effectively develop humane personal contact with professionals and support them must be adopted in the future.

Leaders were not agreed on the significance of their constant presence in the workplace. They emphasised effortless accessibility of leaders as part of meaningful presence. They perceived that they should be more attentive to staying calm in stressful future situations and sustaining both trust and hope in work communities. In addition, they noted that people learn and evolve through their interactions; therefore, accessibility to and positive modelling examples by leaders are needed.

The analysis showed that transparent communication is needed to enhance authenticity in encounters. According to leaders, transparent communication places great importance on their conversation, open dialogue, genuine hearing and responsiveness:

Genuine listening […] Not just listening but hearing what they [professionals] want to say […] and reacting on propositions of professionals. (Interviewee 8)

Further, our findings suggest that developing strong self-leadership includes the self-awareness and resilience of leaders and prioritising their own time and tasks. Leaders perceived that being self-aware requires self-reflection, which allows them to renew and modernise their own leadership styles. Leaders need to constantly revise their leadership knowledge and accept the need to continue their professional development. Moreover, resilience is needed to adapt to changes in healthcare. In addition, leaders need to have the courage to engage in innovative thinking and make critical considerations about established practices – in other words, the courage to disclose weaknesses in operations and to try different procedures and approaches. The analysis also shows that prioritising one’s own time and tasks to release time for professionals will be crucial in the future. One leader explained the need for revising education as follows:

Old traditions of leading are stuck in organisations. With further education, leaders could see new things and ways to lead and notice that there is a lot that can be achieved by them. (Interviewee 16)

Analysis revealed that enhancing agility in an organisation requires flexible leading of complexity, leading with knowledge within large entities and enhancing information flow. Flexible leading of complexity includes anticipation of what will occur in the future, agile responsiveness and the competence to meet the different demands in the changing context of healthcare. Leaders perceived that anticipation of the future means strategic thinking, proactive planning and having constant foresight. Agile responsiveness includes a more sensible and faster reaction to different situations, including implementation of changes. One participant noted this:

Unit leaders can’t just wait and see what’s happening. They must be able to conduct and process things faster. (Interviewee 19)

The competence to lead when encountering the different demands involved in healthcare requires skills to ensure the quality of nursing and flexible leadership of human resources, including rotation of professionals and competence management. Leaders underlined the acceleration of changes and the importance of changing leadership competencies in the future.

Leading with knowledge within large entities requires circumspect knowledge; in other words, leaders need to be constantly updated and aware of facts and circumstances affecting planning and decision-making in complex environments. The ability to perceive the impacts of decisions within large entities is also needed. Future evidence-based leadership requires constant updating and reviewing of knowledge.

According to the interviewees, the pandemic highlighted the importance of enhancing information flow as part of improving agility in organisations. Enhancing information flow requires the development of both functional information systems and adequate information and strengthening the information competencies of leaders. According to the leaders’ perceptions, digital information channels that were initiated and used during the pandemic will continue to advance future information sharing as they allow broader participation and recording of information events. In addition, our analysis indicates that for future information to be adequate, it must be current, truthful and explicit. Therefore, to reduce bias, leaders noted that formal information transitioning through the organisation will be required.

## Discussion

This study produced information about nurse leaders’ perceptions of future leadership in the post-pandemic era in hospital settings. Our findings highlight a clear need to actively enhance digitalisation in healthcare. Implementing digitalisation in leadership provides possibilities to increase efficiency in organisations. One of the most significant requirements related to digitalisation is enhancing leaders’ digital competencies. The possibilities of digitalisation have also been recognised in prior studies (Dirani *et al.*, 2020; Freysteinson *et al.*, 2021; Nurmeksela *et al.*, 2021; Pihlainen *et al.*, 2019). According to Gjellebæk *et al.* (2020), the development and use of digital solutions require enhancing the knowledge and competence of both leaders and professionals.

Our study revealed that remote leadership offers new possibilities, especially when leading large and divergent units. There is also a need to develop a hybrid working culture for future leadership. On the other hand, Ameel *et al.* (2022) found that, although remote leadership enhances efficiency, the absence of leaders might decrease the well-being of professionals.

The development of sustainable working conditions is perceived as pivotal to the success of future leadership. Leaders underscored the importance of enhancing the appeal of the nursing profession and competence in recruiting and leading new generations. The need to understand the diverse needs of younger professionals and change their own leadership style was also recognised. Prior studies have recognised the diverse needs of different generations of professionals (Pihlainen *et al.*, 2019; Nurmeksela *et al.*, 2021); however, there is limited knowledge of the impacts of different leadership styles on younger generations’ work engagement.

The results of our study show that the reputation of the nursing profession, leaders and work communities must be improved to increase nursing and organisational appeal. Moreover, the development of sustainable working conditions requires increasing well-being at work. Also, Nurmeksela *et al.* (2021) indicated that the well-being of professionals requires more attention in the future to increase the attractiveness of nursing. Furthermore, our findings are similar to those of prior studies concerning the increasing need to focus on the well-being of healthcare professionals in the post-pandemic era (Dirani *et al.*, 2020; Morse and Warshawsky, 2021; Okoli *et al.*, 2022).

Our study also revealed that moving smoothly to the post-pandemic era requires completely recovering from the pandemic. The change to a new normal needs to be led, like any other change, by surveying the best possible practices needed in the future. The results also show that active restructuring of operations caused by the pandemic will contribute to recovering and moving into the next era. By arranging the possibility of communally reflecting on the experiences and negative affections caused by the pandemic, leaders will be able to relieve the psychological burden that professionals accrued.

Anticipation of future crises is perceived as pivotal, and this includes the combination of strategic procedures based on the experiences of the pandemic and strengthening competence in crisis leadership. These findings are similar to prior findings that stressed the need to survey the lessons learned from the pandemic (Freysteinson *et al.*, 2021; Morse and Warshawsky, 2021).

According to our results, dissolution of traditional regimes of organisation by strengthening interaction within the organisation and environment will be highly important in the allocation of scarce resources in the future. In addition, using shared leadership will be needed in the future to decrease the hierarchy in hospital organisations. Nurmeksela *et al.* (2021) have also noted that more authentic multidisciplinary cooperation across traditional regimes will be enhanced in the future. Shared leadership has been found to be a possibly valuable leadership style for the future; however, there are and will continue to be challenges in implementing it in hospital settings because of factors such as blurred responsibilities and the challenge of leading strong professions (Pihlainen *et al.*, 2019).

Our results present a valuable contribution by indicating the importance of enhancing flexibility to increase positive encounters in leadership, develop strong self-leadership and enhance agility in organisations. The ability to authentically respond to and support the different needs of professionals while maintaining clarity and assertiveness in leadership is required to ensure leaders’ meaningful presence. Our analysis shows that leaders highly value transparency in communication and conversational leadership. With authentic, genuine listening, leaders can recognise the different needs of professionals and meet those needs through their operations and their own leadership. The importance of leaders’ communication skills has also been clearly recognised in prior studies describing future leadership (Pihlainen *et al.*, 2019) and leadership in the post-pandemic era (Dirani *et al.*, 2020).

Leaders’ self-leadership skills, which underline the need for flexibility, are perceived as necessary in the future. Flexible, self-aware leaders can acknowledge and accept the need for change, particularly in their own leadership. Resilience enhances the ability to accept those changes and provides the courage to point out possible weaknesses in operations. These findings add knowledge to that of prior studies describing the essential characteristics of future leaders, such as self-confidence (Sanford and Janney, 2019), courage to act differently (Pihlainen *et al.*, 2019; Sanford and Janney, 2019), flexibility, responsiveness and prioritising skills (Pihlainen *et al.*, 2019).

Our results indicate that flexibility in leadership enhances agility in organisations. Leading in rapidly changing situations in complex environments, simultaneously anticipating the future and meeting the different demands of healthcare requires flexibility. According to our analysis, the pandemic emphasised the importance of anticipating future events. Nurmeksela *et al.* (2021) also stated that future leadership will be more forward-looking. As in our study, strategic leadership has often been found to be related to anticipation. According to Pihlainen *et al.* (2019), Finnish experts prefer goal orientation over strategic thinking in future leadership because of the challenges in understanding and implementing substantial strategies.

Our results show that successfully addressing different demands for future healthcare will require greater flexibility in leadership. Leaders must be able to constantly provide viable healthcare solutions in their work by simultaneously managing the quality of nursing, flexibility in human resource leadership and acceleration of changes. Additionally, our results clearly indicate the need for change in leadership competencies, which was also recognised in prior studies (Pihlainen *et al.*, 2019; Sanford and Janney, 2019: Vasset *et al.*, 2023).

Our study shows that leading large healthcare entities requires competencies in leading with knowledge, including evidence-based leadership and the ability to be broad-minded and willing to constantly update knowledge. Leaders recommended that information flow should be developed by ensuring the adequacy of information and developing functional and comprehensive information systems by using digitalisation. Additionally, strengthening leaders’ information competencies has been recognised in prior studies (Okoli *et al.*, 2022; Pihlainen *et al.*, 2019). With the most correct and updated information, post-pandemic leaders can support the sense-making and well-being of professionals (Dirani *et al.*, 2020).

The findings of this study indicate that the essence of future leadership is strongly connected with the importance of human leadership skills, such as flexibility in interaction and cooperation skills, thus deviating from prior studies that have highlighted financial skills as an essential part of future leadership (Dirani *et al.*, 2020; Nurmeksela *et al.*, 2020; Pihlainen *et al.*, 2019; Sanford and Janney, 2019). Our study emphasises the meaningful presence of leaders, including authenticity in encounters, similar to how Sanford and Janney (2019) highlighted the need for executive presence, showing confidence and good decision-making skills in future leadership.

### Study limitations

This study has some limitations. This is a single-centre study, so the results may not be transferable to other hospitals or countries. Only one author was responsible for data analysis, although it was discussed with the research team. Preconceptions of the phenomenon were possible because one author had worked as an assistant head nurse during the pandemic. This qualitative interview study might also have been subject to social desirability bias.

## Conclusions and implications

The results demonstrated that future leadership in hospital settings will be determined by various challenges, such as use of digitalisation, maintaining sufficient supply of professionals and well-being of professionals and constantly reducing resources. These challenges are complex and require critical evaluation and changes to traditional leadership. In the future, leaders’ will be required to rationalise the use of time and resources to develop working culture and leadership.

Nurse leaders need to gain the competencies required to lead the next generation and increase the appeal of nursing. Flexibility and more authentic human leadership styles can provide the foundation for new kinds of leadership, better work conditions and more meaningful work. Nurse leaders’ competencies in digital, crisis and human leadership, as well as in anticipating change signals in their environments, should be strengthened and included in leadership education for the future. Healthcare organisations should provide continuous and proactive education for nurse leaders to strengthen their competencies and reflect on their own leadership style. Therefore, studies are needed to examine the competencies needed to anticipate future leadership and lead the next generation in changing, complex hospital environments by using mixed methods and intervention studies.

## Figures and Tables

**Table 1.** Participants’ characteristics (*n* = 20)

Variable	f
*Gender*	
Female	19
Male	1
*Educational level*	
College degree	4
Bachelor's degree	7
Master's university degree or higher	9
*Work task (position)*	
Frontline nurse leader^a^	16
Middle management position	4
	mean
Age (years)	51.0^b^
Leadership experience (years)	10.3^c^

Notes: ^a^ head nurses and assistant head nurses; ^b^SD: 7.3; min, max: 37.0–63.0; ^c^SD: 7.5; min, max: 1.0–29.0

**Source:** Authors’ own work

**Table 2.** Nurse leaders’ perceptions of future leadership in hospital settings in the post-pandemic era

Subcategories (*n* = 26)	Categories (*n* = 11)	Main categories (*n* = 5)
Possibilities of implementing digitalisation to leadership Resources needed for implementing digitalisation	Using digitalisation in leadership	Digitalisation and hybrid working culture
Possibilities of adoption of remote leadership	Development of hybrid working culture in leadership	
Challenges of hybrid work in leadership		
Improving work conditions	Enhancing work engagement of professionals	Development of sustainable working conditions
Competence in leading new generations		
Increasing appreciation of professionals		
Supplying constant support for professionals’ well-being	Increasing well-being at work	
Supporting the well-being of leaders		
Change to a new normal	Recovering from the pandemic	Moving smoothly to the post-pandemic era
Assessing and restructuring operations caused by the pandemic		
Reflecting on the pandemic within the organisation		
Combination of strategical procedures for dealing with future crises	Anticipating and preparing for the future crises	
Strengthening crisis leadership competence		
Restructuring hierarchy in the organisation	Using shared leadership	Dissolution of traditional regimes of organisational structure
Sharing responsibilities of leaders		
Improving communality in work communities	Enhancing cooperation in the organisation	
Strengthening interaction within the organisation and environment		
Enhancing meaningful presence of leaders	Increasing authentic encounters in leadership	Flexibility in leadership
Transparent communication		
Self-awareness of leaders	Developing strong self-leadership	
Resilience of leaders		
Prioritising one’s own time and tasks		
Flexible leading of complexity Leading with knowledge within large entities Enhancing information flow	Enhancing agility in organisation	
	
	

**Source:** Authors’ own work

## Supplementary material

The supplementary material for this article can be found online.
