# Supplementary material for: Nurse leaders’ perceptions of future leadership in hospital settings in the post-pandemic era: a qualitative descriptive study
Source: Leadersh Health Serv (Bradf Engl). 2023 Sep 28;37(5):33–48. doi: 10.1108/LHS-05-2023-0032 (PMC10868662; doi:10.1108/LHS-05-2023-0032)
Supplement: Supplementary file 1 [file leadershhealthserv-37-0033-s001.docx]

Supplementary table I. Example of the analysis process in the main category of development of sustainable working conditions

| Open codes | Subcategories | Categories | | | Main category | |
| --- | --- | --- | --- | --- | --- | --- |
| Intention to leave among nurses is concerning (Interviewee 9) | Improving work conditions (27)* | Enhancing work engagement of professionals | | | Development of sustainable working conditions | |
| Competencies in maintaining sufficiency are needed (Interviewee 14) |  |  | | |  | |
| People want to leave even though they like the work (Interviewee 9) |  |  | | |  | |
| Enhancing meaningful work is important (Interviewee 14) |  |  | | |  | |
| Job descriptions should be reassessed (Interviewee 1) |  |  | | |  | |
| Attractiveness of nursing must be enhanced (Interviewee 9) |  |  | | |  | |
| Decreasing negative reputation of nursing is important (Interviewee 8) |  |  | | |  | |
| Changes in leadership style must be made (Interviewee 1) |  |  | | |  | |
| Human resource policies should be developed (Interviewee 14) |  |  | | |  | |
| Needs of new generations should be recognised (Interviewee 16) | Competence on leading new generations (15) |  | | |  | |
| Young people don’t commit to workplaces like before (Interviewee 14) |  |  | | |  | |
| Need to be open to change when leading new generations (Interviewee 9) |  |  | | |  | |
| Increase of salary might enhance commitment (Interviewee 17) | Increasing appreciation of professionals (12) |  | | |  | |
| Professionals should be more appreciated (Interviewee 9) |  |  | | |  | |
| Fatigue increased when the pandemic subsided (Interviewee 8) | Supplying constant support for professionals’ well-being (29) | Increasing well-being at work | | |  | |
| Supporting well-being of professionals is important (Interviewee 12) |  |  | | |  | |
| Leaders need to focus on professionals’ well-being (Interviewee 16) |  |  | | |  | |
| Mentoring might enhance well-being of leaders (Interviewee 15) | Supporting well-being of leaders (28) |  | | |  | |
| Peer support enhances well-being of leaders (Interviewee 10) |  |  | | |  | |
| Leaders should be offered debriefing after the pandemic (Interviewee 3) |  |  | | |  | |
| *The number of open codes in subcategories are shown in brackets. Source: Authors’ own work. | | |  |  | |  |
